# Turnover among Australian general practitioners: a longitudinal gender analysis

**DOI:** 10.1186/s12960-020-00525-4

**Published:** 2020-12-09

**Authors:** E. Anne Bardoel, Grant Russell, Jenny Advocat, Susan Mayson, Margaret Kay

**Affiliations:** 1 Swinburne Business School, Swinburne University of Technology, Mail H23, Cnr John and Wakefield Streets, PO Box 218, Hawthorn, VIC 3122 Australia; 2 Monash University, Melbourne, Australia; 3 University of Queensland, Brisbane, Australia

**Keywords:** General practitioners, Turnover, Gender role theory, Unfolding theory of turnover, Family-friendly

## Abstract

**Background:**

Little is known about gender differences in general practitioner (GP) turnover. It is important to understand potential divergence given both the feminization of the Australian GP workforce and projected shortages of GPs.

**Objective:**

There is increasing evidence that national health outcomes are related to the extent to which health care systems incorporate high quality primary care. Quality primary care is, in turn reliant on a stable general practice (GP) workforce. With the increasing feminization of medical schools, we sought to identify correlates of turnover in the GP workforce, separately for women and men, focusing particularly on part-time employment and child-rearing, and distinguishing effects related to either planned or unplanned turnover.

**Methods:**

Annual responses from cohorts of at least 1900 women GPs and 2000 men GPs are used for up to eight waves of the Medicine in Australia—Balancing Employment and Life (MABEL) longitudinal survey of doctors. Descriptive and bivariate correlations are provided. Random effects ordered logit is applied to dependent variables for turnover intentions measuring intent to “leave direct care” or “leave medicine”. A behavioral measure of turnover is used in random effects logit regressions, with the exclusion or inclusion of the confounding intentions variables revealing correlates of unplanned or planned turnover.

**Results:**

Part-time employment is associated with turnover intentions among both women (84% or 94% increase in the odds ratios or ORs) and particularly men (414% or 672%), and with actual turnover for women (150% or 49%) and for men (160% or 107%). Women GPs engage in more unplanned turnover than men: they are 85% more likely to engage in turnover after controlling for intentions. Unplanned turnover is concentrated among women below 40 years of age and with young children, even though both groups report below average turnover intentions.

**Conclusion:**

Although further studies are needed to identify specific factors associated with GP turnover among women, the analysis highlights the need to focus on women GPs who are either young or have young children. Given the substantial personal and social investment required to produce GPs, it is wasteful to lose so many young women early in their careers.

## Introduction

General practitioners (GPs) leaving the profession is costly not only in terms of societal investments in training new GPs [1], but also because of the significant loss of investments of time and money by young GPs. Strategies to reduce turnover could also play a role in alleviating projected workforce shortages of GPs [2]. Degen et al.’s systematic review of 17 studies of GP turnover intentions [3] found that women were more likely than men to report family reasons or workplace harassment as reasons for turnover intentions. They also called for further research focused on understanding gender differences in relation to intention to leave and actually leaving general practice.

This study aims to help fill this gap by investigating longitudinal data on cohorts of GPs to ascertain differences and similarities across women and men in the correlates of turnover and turnover intentions, focusing particularly on the roles of part-time employment and young children, and their relationship to planned and unplanned turnover.

## Background

Turnover among GPs is a problem if, as in Australia, workforce shortages are predicted [2]. This problem is compounded when an aging population exists which expands the need for care [4], or if GPs leave practice prior to retirement age [5], which involves the loss of significant investments in training.

Turnover and turnover intentions vary across women and men [3, 6]. This factor is relevant because most young GPs in Australia are women. Every year since 2007 (the first year for which data are available), women have comprised a majority of Australian medical students [7] and by 2017, most GPs (51%) were women [8].

Underlying the feminization of GPs may lie in part a belief among young women that the career is family-friendly. That possibility appears in qualitative Australian research suggesting that some young women enter the field, and work part-time or with flexible hours, to balance caring for children with paid employment [3].

Part-time employment may also influence or be related to turnover. As of 2011, 35% of Australian female GPs worked part-time and 13% of male GPs also worked part-time [9]. Using a random sample of Australian households, Booth and van Ours [10] found that women working part-time are typically more satisfied with their work hours than their full-time counterparts, but that the relationship was reversed for men working part-time. Fuchs Epstein and colleagues [11] argue that part-time work may be marginalized and this dynamic is more salient to women because of gender stereotyping.

Parenting might also affect turnover and be experienced differently by male and female GPs. A qualitative study of female GPs in Australia revealed substantial conflicts between demanding professional roles and care for children [12]. On the other hand, part-time work should, as many young female GPs suggest [3, 13, 14], facilitate balancing work and family demands.

Substantial research demonstrates that behavioral intentions are strong predictors of behavior [15], and specifically that intentions to leave (turnover intentions) are among the strongest predictors of actual turnover [16–19]. Central to the present analysis is the difference between planned and unplanned (or shock-driven) turnover. Planned and unplanned turnover can be distinguished by the degree to which turnover intentions influence behavior: the stronger the relationship, the more often planned turnover exists while, conversely, the weaker the relationship, the more often unplanned turnover occurs. While planned turnover may be related to long-term dissatisfaction, it also provides an opportunity to behave strategically, marshalling resources for job search or non-employment, while such options may not be available for unplanned turnover.

Other independent variables found to influence GP turnover intentions in prior studies include having a partner or spouse [6], and age, with older GPs more likely to intend turnover [3]. Long work hours may also increase turnover intentions [3]. Unpredictable work hours, often associated with on-call work, might also raise turnover intentions [6]. Russell et al. [20] found higher rates of GP turnover in remote communities of Australia, although Scott et al. [21] found mixed results. Work in a hospital setting has mixed effects on intentions [22, 23]. Australia also introduced experimental integrated health operations, or ‘super clinics’, in 2008 [24], and co-location with other health professionals may also influence turnover. An additional issue is the presence of immigrants in the GP workforce, who generally exhibit above-average turnover rates [25]. Turnover may also be inversely related to income [26].

## Data

The analysis uses a subset of the Medicine in Australia—Balancing Employment and Life (MABEL) longitudinal survey of doctors. Survey details have been previously reported [27]. The first iteration of the survey was distributed in 2008, with written invitations sent to all 54,750 identifiable doctors as listed in the Medical Directory of Australia (https://ampcodirect.com.au). The initial cohort includes 10,498 doctors responding to the Wave 1 survey [21]. Although the cohort was followed for up to 8 waves, there is a substantial attrition and generally low response rates, despite multiple options and incentives to complete the surveys, and top-up samples of new cohorts were added each year.

The first eight waves of the MABEL are utilized with turnover intentions questions asked in waves 1–4, and actual turnover constructed from data collected in waves 2–8. To utilize the longitudinal nature of the data, respondents only appearing in one survey wave are excluded.

The dependent variables for turnover intentions are ascertained by two items scored on a 5-point Likert scale from “strongly disagree” through “strongly agree”. The first concerns “the likelihood that you will leave direct patient care (primary or hospital) within 5 years”, (*leave direct care*), which is the definition used by Degen et al. [3]. The second is “the likelihood that you will leave medical work entirely within 5 years”, (*leave medicine*).

The other dependent variable is a behavioral measure of turnover is developed for this study. Measured *turnover* involves two sequential events: a report of not performing clinical work in one survey wave, followed by a minimum of at least one further wave (up to the maximum available) in which the respondent either continues to report no clinical work or no longer participates in the survey (i.e., never responds again). This measure of *turnover* yields 447 GPs who permanently left clinical work between waves 2 and 7 (inclusive). As a check, 261 respondents were found who reported no clinical work in one wave (for waves 2–5), either no clinical work or no response in the next wave (waves 3–6), but nonetheless responded to the MABEL in the following wave (waves 4–7). None of those respondents reported clinical work in that wave, supporting use of the turnover measure.^1^

While splitting the sample into groups of women and men, the key independent variables of interest here are for part-time employment, and parents of young children. Part-time employment is measured as less than 35 h during a typical workweek [9]. For young children, the variable is measured by the household presence of children less than 5 years of age and with no older children; the latter restriction eliminates parents who have likely discovered resources to help them integrate their child-rearing and GP work responsibilities.^2^

Other independent variables include having a partner or spouse. Age is measured categorically in the MABEL, so the values are mid-pointed. Researchers typically categorize long work hours as above 50 h per week [28], as is also done here. A measure of unpredictable work hours is generated from responses to, “The hours I work are unpredictable” (5-point Likert scale for agreement). Relevant variables for work in either an urban location (“major city”) or outback (“outer regional/remote/very remote”) are constructed from the Australian Statistical Geographic Classification code for the GPs main place of work. Work in a hospital setting utilizes responses to, “Do you currently work in a hospital?” (yes/no responses). The measure of co-location is from responses to: “Is your current main practice co-located with other health or welfare professionals” (yes/no responses). Immigrants are proxied by an item on non-citizen status. Given only young children are accounted for in the key independent variables, a variable for the total number of dependent children in the household is also included, with income measured by dummy variables for GP income below the 25th percentile or above the 75th percentile for all GPs in each year.^3^

The variables and sample are described in Table 1, with figures reported separately for women and men. According to the first two sets of figures in the table, women are less likely to intend to *leave direct care* or *leave medicine*. *Turnover* is indistinguishable at 1.3% of all observations for women or men, although that figure is for all observations, and 8.4% of the respondents are eventually classified as positive on *turnover*. For the key independent variables, women are over three times as likely to work part-time by either measure, and are half-again more likely to parent young children.

**Table 1:** Characteristics of sample, 2008–2015

	Women	Men
Leave direct care (scale 0–4)^a^	.950	1.39
Leave medicine (scale 0–4)^b^	.761	1.14
Turnover^c^	1.3%	1.3%
Part-time (< 35 h)	36.1%	8.4%
Young child (< 5 years)	9.6%	6.2%
Partner/spouse	83.1%	91.4%
Age < 40	24.3%	11.2%
Age 40 to 49	32.7%	22.6%
Age 50 to 59	31.5%	36.9%
Age 60+	11.5%	29.3%
Hours 35–50	41.8%	33.1%
Hours >50	22.1%	58.6%
Hours vary (scale 0–4)	1.33	1.64
Work city	68.5%	58.6%
Work outback	13.4%	16.9%
Hospital work	19.8%	32.7%
Co-location other health profs.	48.9%	45.2%
Immigrant	9.9%	11.3%
Number of children	1.35	1.20
Hi income (> 75th %)	11.0%	38.9%
Low income (< 25th %)	37.4%	11.8%
Observations	8791	9633
Number of GPs	2040	2133

^a^5046 observations for 1959 women GPs; 5670 observations for 2077 men GPs

^b^5015 observations for 1947 women GPs; 5640 observations for 2055 men GPs

^c^7964 observations for 2040 women GPs; 8733 observations for 2133 men GPs. All figures unweighted

## Methods

Descriptive, bivariate, and multiple logistic regressions are applied to these data. Observations on 2040 women GPs and 2133 men GPs are available for all independent variables (see Table 1), with fewer observations available for the dependent variables; given small cell sizes, discussed next, all available observations are used for each test.

The bivariate analysis uses simple comparisons and correlations, and each statistic is calculated on separate groups of women and men. Since the variables for turnover intentions are categorical but ordered, and the young child, partner, and part-time variables are dichotomous, a Kruskal–Wallis *H* test is used to gauge correlations [29]. Turnover behavior is dichotomous, so a *χ*^2^ test is applied to indicate correlations with the key independent variables.

Testing for whether female GPs working part-time with young children are likely to leave general practice involves very small cell sizes, so testing utilizes four probabilities for women and for men, while limiting the sample to one observation for each GP woman or man (for either the year of *turnover* or the most recent year for no turnover). First is turnover among part-timers with young children; second is turnover among part-timers without young children; third is turnover among full-timers with young children; and fourth is turnover among full-timers without young children.

Additionally, given that overall turnover figures are identical for women and men (see Table 1), it is worth considering an age comparison of the women and men who engaged in turnover. To the extent turnover is either associated with young children or unplanned events among women, then rates of turnover should be higher among young GP women relative to young GP men.

Regression analyses are utilized with the dependent variables being *leave direct care* or *leave medicine* for intentions, and *turnover* for behavior. For the first two variables, intentions from waves 1 through 4 are used as the dependent variables in regressions. Given the Likert-scaling of the variables for intentions, a random effects ordered logistic regression with standard errors clustered on individual GPs is applied [18]. Odds ratios (ORs) are reported for effect sizes, so 2.0 implies a doubling of the dependent variable for a one-unit increase in the independent variable, and 0.5 implies a halving of the dependent variable for a similar increase in the independent variable. For behavior, *turnover* is a dichotomous event, so a random effects (simple) logistic regression with standard errors clustered on GPs is used [30]. Note also that, since anyone reporting permanent retirement is not asked to complete most other MABEL survey questions, the actual turnover measure is carried backward one wave (i.e., the one-period lead value of actual turnover is used) so non-missing independent variables are available for the analysis. All regressions are performed separately for women and men.

Regression analyses initially use the intentions variables along with all independent variables. These results can shed light on whether results found in the simple correlations hold in the presence of other controls.

Regressions for *turnover* are presented both without and with the *leave direct care* and *leave medicine* as confounding variables. Given the latter are only available for waves 1–4, and measured *turnover* can occur from waves 2–6, the most recent response to the intentions variables is carried forward into future observations. Regression results with unadjusted ORs are available as additional information. Stata, version 15.1 (StataCorp, College Station, Tex, USA) is used for the analysis (see Appendices 1 and 2).

## Results

Differences in turnover intentions and behavior, by gender, and by key independent variables, are reported in Table 2. Whether measured by intentions or by behavior, and across women and men, part-time work is associated with elevated turnover intentions and behavior. These results support the notion that part-time work contributes to turnover intentions and behavior regardless of gender, and these correlations are stronger for men than for women.

**Table 2:** Turnover intentions and behavior by gender and key controls (*p* values)

Independent variables	Leave direct care, women	Leave direct care, men	Leave medicine, women	Leave medicine, men	Turnover, women	Turnover, men
Part-time (< 35 h)	1.02	2.68	.867	2.57	3.0%	8.8%
Full-time (≥ 35 h)	.931	1.29	.715	1.03	1.2%	1.3%
Test for diff	(.031)	(.000)	(.000)	(.000)	(.000)	(.000)
Young chd (< 5 years)	.557	.617	.373	.477	2.2%	0.0%
No yng chd (< 5 years)	1.02	1.49	.825	1.23	1.9%	2.2%
Test for diff	(.000)	(.000)	(.000)	(.000)	(.603)	(.001)
Partner	.961	1.41	.768	1.17	1.7%	1.6%
No partner	.960	1.49	.766	1.19	1.2%	1.9%
Test for diff	(.878)	(.109)	(.917)	(.593)	(.141)	(.518)

Test statistics are Kruskal–Wallis *χ*^2^ for *Leave direct care* and *Leave medicine*, and simple *χ*^2^ for difference of proportions for *Turnover*

Turnover intentions are lower among mothers of young children compared to other women GPs. Nonetheless, women with children under 5 years of age are significantly more likely to engage in turnover.

For the four categories for young children and part-time variables (Table 3), the highest rates of turnover are for GPs who are mothers of young children working part-time (14%).

**Table 3:** Turnover by gender, the roles of part-time work and young children, % (number)

	Women	Men
Part-time w. yng. child	14.0% (57)	0.0% (6)
Part-time no yng. child	7.3% (731)	31.7% (224)
Full-time w. yng child	8.2% (134)	2.2% (90)
Full-time no yng. child	4.0% (1236)	4.6% (1933)

Considering age and turnover, the age distribution of turnover among female GPs places the median at age 47, with 83 of 171 turnover events (48.5%) associated with that age or below. Among the male GPs, only 38 of 195 turnover events occur for age 47 or younger (19.5%), with a majority of turnover occurring above age 67.

ORs, along with *p*-values and 95% confidence intervals (CIs), from the regressions for intentions are reported in Table 4, which demonstrates that part-time work is positively and significantly associated with turnover intentions, by either measure, and for women and men. Partnered GP women are slightly more likely to intend leaving direct care, youth is associated with low turnover intentions (relative to the omitted group of GPs age 60 years and above), variable hours are positively associated with intentions, location in a city is mildly associated with lesser turnover intentions, and location in the outback is related to lower intentions to leave medicine among women. Finally, low income is associated with elevated turnover intentions, consistent with economic models.

**Table 4:** Turnover intentions, ordered logistic regressions, ORs (*p* values) [95% CI]

Independent variables	Leave direct care, women	Leave direct care, men	Leave medicine, women	Leave medicine, men
Part-time (< 35 h)	1.84	5.14	1.94	7.62
	(0.00)	(0.00)	(0.00)	(0.00)
	[1.34–2.55]	[3.03–8.73]	[1.38–2.73]	[4.33–13.39]
Young child (< 5 years)	0.82	0.69	0.70	0.79
	(0.32)	(0.06)	(0.06	(0.23)
	[0.56–1.21]	[0.47–1.01]	[0.48–1.01]	[0.53–1.16]
Partner/spouse	1.35	0.84	1.32	1.02
	(0.07)	(0.38)	(0.12)	(0.90)
	[0.98–1.86]	[0.58–1.23]	[0.93–1.86]	[0.70–1.50]
Age < 40	0.02	0.02	0.01	0.01
	(0.00)	(0.00)	(0.00)	(0.00)
	[0.01–0.03]	[0.01–0.03]	[0.01–0.02]	[0.01–0.02]
Age 40 to 49	0.03	0.03	0.02	0.03
	(0.00)	(0.00)	(0.00)	(0.00)
	[0.02–0.05]	[0.02–0.05]	[0.01–0.03]	[0.02–0.05]
Age 50 to 59	0.09	0.09	0.07	0.08
	(0.00)	(0.00)	(0.00)	(0.00)
	[0.06–0.15]	[0.06–0.13]	[0.05–0.12]	[0.05–0.12]
Hours > 50	1.00	0.69	0.85	0.67
	(1.00)	(0.02)	(0.41)	(0.01)
	[0.69–1.45]	[0.51–0.93]	[0.58–1.24]	[0.49–0.91]
Hours vary	1.33	1.44	1.31	1.36
	(0.00)	(0.00)	(0.00)	(0.00)
	[1.21–1.47]	[1.32–1.58]	[1.17–1.45]	[1.24–1.50]
Work city	0.77	0.72	0.69	0.82
	(0.10)	(0.03)	(0.03)	(0.21)
	[0.56–1.05]	[0.54–0.98]	[0.50–0.96]	[0.60–1.12]
Work outback	0.85	0.91	0.64	0.95
	(0.47)	(0.62)	(0.06)	(0.80)
	[0.55–1.32]	[0.63–1.32]	[0.41–1.01]	[0.65–1.40]
Hospital work	0.87	0.76	0.73	0.73
	(0.30)	(0.03)	(0.03)	(0.02)
	[0.66–1.13]	[0.60–0.97]	[0.55–0.97]	[0.57–0.94]
Co-location	0.94	1.09	0.86	0.99
	(0.50)	(0.34)	(0.14)	(0.92)
	[0.77–1.13]	[0.91–1.31]	[0.70–1.05]	[0.82–1.20]
Immigrant	0.89	0.97	1.21	1.00
	(0.65)	(0.90)	(0.45)	(0.98)
	[0.55–1.45]	[0.64–1.49]	[0.74–2.01]	[0.64–1.56]
Number of children	0.63	0.69	0.63	0.70
	(0.00)	(0.00)	(0.00)	(0.00)
	[0.56–0.71]	[0.62–0.78]	[0.55–0.71]	[0.62–0.78]
High income	0.89	0.98	0.91	0.96
	(0.48)	(0.84)	(0.59)	(0.67)
	[0.65–1.23]	[0.82–1.18]	[0.65–1.28]	[0.78–1.17]
Low income	1.21	1.42	1.26	1.39
	(0.07)	(0.01)	(0.04)	(0.02)
	[0.98–1.50]	[1.08–1.88]	[1.01–1.57]	[1.05–1.85]
Observations	5046	5670	5015	5640
Number of GPs	1972	2090	1970	2088
*χ*^2^	432.3	772.5	495.9	764.5
	(.000)	(.000)	(.000)	(.000)

Results for the test of predicting turnover with only intentions and a woman variable find both intentions coefficients are positive, with *leave direct care* significant (OR = 1.65, *p* < 0.001), and *leave medicine* insignificant (OR = 1.26, *p* < 0.106). The woman coefficient is positive and significant (OR = 1.85, *p* < 0.003), which suggests that women GPs are approximately 85% more likely than men to turnover after controlling for intentions.

Results for extended turnover regressions are provided in Table 5, noting that the left-most column for women or men excludes, and the right-most column includes the confounding intentions variables. Considering results absent the intentions variables, both connect part-time employment with turnover. The coefficients for women and young child are insignificant. However, the women’s GP turnover regression generally has low predictive power (i.e., insignificant *χ*^2^ statistic), which is not the case for men, a result consistent with greater unintended turnover among women. For other control variables, young GPs are less likely to engage in turnover, while men in co-location facilities or with low incomes are more likely to turnover, and women with a large number of children are less likely to turnover.

**Table 5:** Turnover, logistic regressions, ORs (*p* values) [95% CI]

Independent variables	Turnover, no intent, women	Turnover, w. intent, women	Turnover, no intent, men	Turnover, w intent, men
Part-time (< 35 h)	2.50	1.49	2.60	2.07
	(0.10)	(0.25)	(0.06)	(0.06)
	[0.84–7.37]	[0.75–2.94]	[0.94–7.18]	[0.97–4.42]
Young child (< 5 years)	1.42	1.37	n.o	n.o
	(0.55)	(0.45)		
	[0.46–4.42]	[0.60–3.12]		
Partner/spouse	1.66	1.29	0.81	0.64
	(0.39)	(0.54)	(0.62)	(0.37)
	[0.52–5.27]	[0.58–2.87]	[0.35–1.88]	[0.25–1.67]
Age < 40	0.83	5.45	0.22	0.44
	(0.76)	(0.00)	(0.07)	(0.42)
	[0.25–2.72]	[2.13–13.96]	[0.04–1.15]	[0.06–3.26]
Age 40 to 49	0.29	2.12	0.11	0.41
	(0.09)	(0.22)	(0.01)	(0.22)
	[0.07–1.23]	[0.64–7.01]	[0.02–0.54]	[0.10–1.74]
Age 50 to 59	0.27	1.06	0.40	0.83
	(0.02)	(0.89)	(0.01)	(0.68)
	[0.09–0.85]	[0.44–2.58]	[0.20–0.82]	[0.35–1.99]
Hours > 50	0.67	0.40	1.04	1.22
	(0.50)	(0.08)	(0.92)	(0.63)
	[0.21–2.16]	[0.15–1.10]	[0.52–2.08]	[0.53–2.82]
Hours vary	1.07	1.08	1.06	1.08
	(0.74)	(0.65)	(0.61)	(0.53)
	[0.73–1.57]	[0.78–1.48]	[0.84–1.35]	[0.86–1.35]
Work city	1.76	1.40	1.27	1.12
	(0.33)	(0.37)	(0.58)	(0.78)
	[0.57–5.48]	[0.66–2.97]	[0.54–2.98]	[0.50–2.51]
Work outback	1.73	1.16	1.48	1.06
	(0.45)	(0.78)	(0.34)	(0.91)
	[0.42–7.12]	[0.40–3.37]	[0.66–3.32]	[0.39–2.90]
Hospital	0.85	0.96	0.87	1.30
	(0.75)	(0.90)	(0.73)	(0.49)
	[0.30–2.36]	[0.46–1.98]	[0.41–1.87]	[0.61–2.74]
Co-location	1.53	1.38	1.55	1.68
	(0.22)	(0.21)	(0.06)	(0.09)
	[0.78–3.00]	[0.83–2.30]	[0.99–2.44]	[0.92–3.07]
Immigrant	1.42	1.17	1.37	2.69
	(0.66)	(0.75)	(0.64)	(0.05)
	[0.30–6.73]	[0.43–3.18]	[0.37–5.13]	[0.98–7.37]
Number of children	0.58	0.72	0.78	0.83
	(0.03)	(0.07)	(0.14)	(0.40)
	[0.35–0.94]	[0.50–1.03]	[0.55–1.09]	[0.55–1.27]
Hi income	0.90	1.28	0.68	0.54
	(0.87)	(0.68)	(0.30)	(0.21)
	[0.23–3.42]	[0.41–4.02]	[0.33–1.40]	[0.21–1.41]
Low income	1.44	1.27	2.98	2.41
	(0.35)	(0.44)	(0.00)	(0.01)
	[0.67–3.11]	[0.70–2.30]	[1.57–5.65]	[1.28–4.52]
Leave direct care		1.59		1.62
		(0.03)		(0.09)
		[1.06–2.39]		[0.92–2.85]
Leave medicine		1.28		1.23
		(0.27)		(0.35)
		[0.82–		[0.79–1.93]
Observations	5912	4270	6015	4355
Number of GPs	1823	1751	1817	1730
*χ*^2^	22.60	99.15***	60.10***	73.43***
	(0.13)	(0.00)	(0.00)	(0.00)

*n.o.* no observations

***p < 0.01

Comparing the results which include intentions, for women, the *leave direct care* effect is positive (OR > 1). The part-time OR loses significance, although it remains above ‘1’, with the value implying a 49% increase in turnover associated with part-time work. On net, these results suggest part-time work is mainly but not entirely associated with planned turnover for women. The number of children result remains, suggesting that having a large number of dependent children is associated with low turnover regardless of changing circumstances. The age < 40 effect becomes both large and significant once intentions are included. That finding suggests that the high turnover found among young women, described in the age breakdown above, is associated with unplanned turnover.

Turning to men, while the part-time coefficient declines, it remains positive once intentions are included, suggesting a mix of planned and unplanned turnover is associated with part-time work for men. Similarly, co-location and low income remain significant, associating them with unplanned turnover. An OR which rises to become significant is for immigrant men, which implies that GP turnover among immigrant men is typically unplanned.

## Discussion

Part-time work was a significant predictor of turnover intentions and actual turnover for both men and women. Intentions to leave and actual turnover are higher among men working part-time relative to comparable women, consistent with Booth and van Ours findings [10]. Researchers have pointed to work cultures often devaluing part-timers [11], so it is possible that GP men working part-time generate adverse reactions among patients and colleagues so that part-time work is not currently tenable for many GP men.

Part-time work was once confined primarily to entry-level or hourly-wage positions but as we can see it is also slowly becoming the province of professionals such as GPs, as more parents, young people and retirees seek flexible schedules. The results of this study point to a need for developing initiatives for developing quality part-time work for GPs which take a mutual gains approach to GP and workplace outcomes. This could potentially include career paths for part-time GPs to partnership, access to the same training opportunities as full-time GPs, and part-time work that is actually part-time work and does not mean being paid 50% but doing an 80% work week.

A concerning finding is that GP women with young children are less likely to intend to leave, but actually end up more likely to do so, suggesting that turnover among GP mothers of young children is often unplanned. It is possible that some young women planning to become GPs in order to balance work and commitments to child-rearing [3, 13, 14], find that GP work encroaches substantially on child-rearing time; other factors such as ill treatment or attractive career alternatives might be involved, but the evidence does not suggest that a significant portion of young women become GPs while planning to turnover during child-rearing.

Unplanned turnover among women GPs is also concentrated among those below 40 years of age. This finding implies that the years of schooling and training these women devoted to the field were often tossed aside after a few short years of practice. For society, these losses to the GP labor force are extremely costly, as these women might have practiced for two or three additional decades before new investments in replacement GPs were needed.

Several potential sources of bias may affect the analysis. First, respondents to the MABEL data tend to be female, younger, and come from non-metropolitan areas relative to the entire population of Australian GPs [27]. Second, as suggested above, turnover among young women GPs could be related to ill treatment or attractive career alternatives which are not measured in this study. Third, it is possible that some individuals classified as exhibiting turnover later return to practice.

A strength in the present study is it uses longitudinal data, a large sample, and combines subjective turnover intentions variables with a behavioral measure of actual turnover. However, qualitative studies are needed to understand the potential factors affecting GPs’ intention to leave their jobs. Such studies could shed light on how part-time work could be made a more attractive option for men and women GPs, and explore why many young women GPs engage in unplanned turnover.

## Conclusion

Research is needed to understand why the intentions to leave and actual turnover are higher among men working part-time relative to comparable women. Further studies are also needed to identify specific factors associated with unplanned turnover among women GPs and the analysis highlights the need to focus on women GPs who are either young or have young children. Given the substantial personal and social investment required to produce GPs, it is wasteful to lose so many young women early in their careers.

## Data Availability

The dataset analyzed during the current study are available in the MABEL repository, https://melbourneinstitute.unimelb.edu.au/mabel/for-researchers/data. Applications to have access to the data will be considered by the MABEL research team in its absolute discretion and applicants will be notified of their success.

## Appendix 1

See Table 6.

**Table 6:** Turnover intentions, ordered logistic regressions, unadjusted ORs (*p* values) [95% CI]

Independent variables	Leave direct care, women	Leave direct care, men	Leave medicine, women	Leave medicine, men
Part-time (< 35 h)	1.40	51.0	1.72	97.04
	(0.04)	(0.00)	(0.00)	(0.00)
	[1.02–1.92]	[27.4–94.7]	[1.22–2.44]	[50.0–191.8]
Young child (< 5 years)	0.47	0.35	0.34	0.34
	(0.00)	(0.00)	(0.00)	(0.00)
	[0.33–0.66]	[0.23–0.53]	[0.24–0.48]	[0.22–0.53]
Partner/spouse	1.06	0.88	1.00	1.22
	(0.75)	(0.49)	(0.99)	(0.33)
	[0.75–1.48]	[0.61–1.27]	[0.69–1.45]	[0.82–1.80]
Age < 40	0.40	0.18	0.26	0.13
	(0.00)	(0.00)	(0.00)	(0.00)
	[0.30–0.52]	[0.12–0.28]	[0.19–0.35]	[0.08–0.22]
Age 40 to 49	0.43	0.34	0.42	0.28
	(0.00)	(0.00)	(0.00)	(0.00)
	[0.34–0.53]	[0.25–0.45]	[0.33–0.54]	[0.21–0.39]
Age 50 to 59	1.65	0.56	1.65	0.52
	(0.00)	(0.00)	(0.00)	(0.00)
	[1.33–2.04]	[0.45–0.70]	[1.30–2.10]	[0.41–0.66]
Age 60+	21.1	28.2	29.5	33.6
	(0.00)	(0.00)	(0.00)	(0.00)
	[12.9–34.4]	[19.6–40.7]	[17.7–40.2]	[22.9–49.3]
Hours 35–50	0.65	0.80	0.62	0.76
	(0.01)	(0.25)	(0.01)	(0.20)
	[0.48–0.88]	[0.55–1.17]	[0.45–0.87]	[0.51–1.15]
Hours > 50	1.16	0.28	0.93	0.22
	(0.40)	(0.00)	(0.73)	(0.00)
	[0.82–1.67]	[0.20–0.41]	[0.64–1.37]	[0.15–0.32]
Hours vary	1.33	1.24	1.26	1.15
	(0.00)	(0.00)	(0.00)	(0.00)
	[1.21–1.45]	[1.13–1.35]	[1.14–1.39]	[1.05–1.26]
Work city	1.12	1.29	1.15	1.51
	(0.43)	(0.12)	(0.35)	(0.01)
	[0.85–1.48]	[0.94–1.78]	[0.85–1.56]	[1.09–2.10]
Work outback	0.78	0.77	0.54	0.69
	(0.20)	(0.18)	(0.00)	(0.07)
	[0.53–1.14]	[0.52–1.13]	[0.36–0.82]	[0.47–1.03]
Hospital work	0.87	0.65	0.75	0.57
	(0.26)	(0.00)	(0.03)	(0.00)
	[0.68–1.11]	[0.51–0.83]	[0.57–0.98]	[0.44–0.73]
Co-location	0.91	1.01	0.89	0.96
	(0.32)	(0.87)	(0.26)	(0.68)
	[0.76–1.09]	[0.85–1.21]	[0.73–1.09]	[0.79–1.17]
Immigrant	0.59	0.30	0.68	0.26
	(0.05)	(0.00)	(0.19)	(0.00)
	[0.35–1.00]	[0.18–0.51]	[0.39–1.21]	[0.16–0.47]
Number of children	0.57	0.49	0.56	0.49
	(0.00)	(0.00)	(0.00)	(0.00)
	[0.52–0.64]	[0.44–0.55]	[0.50–0.63]	[0.44–0.56]
High income	0.91	0.73	0.87	0.72
	(0.54)	(0.00)	(0.42)	(0.00)
	[0.66–1.24]	[0.60–0.88]	[0.62–1.22]	[0.58–0.89]
Low income	1.17	2.04	1.23	1.84
	(0.13)	(0.00)	(0.06)	(0.00)
	[0.96–1.44]	[1.52–2.73]	[0.99–1.52]	[1.37–2.48]

## Appendix 2

See Table 7.

**Table 7:** Turnover, logistic regressions, unadjusted ORs (*p* values) [95% CI]

Independent variables	Turnover, women	Turnover, men
Part-time (< 35 h)	n.c	52.56
		(0.00)
		[20.0–137.8]
Young child (< 5 years)	1.35	n.o
	(0.61)	
	[0.52–3.49]	
Partner/spouse	n.c	0.82
		(0.65)
		[0.35–1.01]
Age < 40	1.94	0.03
	(0.20)	(0.00)
	[0.71–5.28]	[0.00–0.31]
Age 40 to 49	0.32	0.43
	(0.01)	(0.00)
	[0.13–0.74]	[0.01–0.17]
Age 50 to 59	0.41	0.18
	(0.04)	(0.00)
	[0.17–0.95]	[0.08–0.41]
Age 60+	15.9	35.8
	(0.00)	(0.00)
	[3.95–64.3]	[17.6–72.7]
Hours 35–50	n.c	0.55
		(0.37)
		[0.15–2.04]
Hours > 50	0.26	0.14
	(0.91)	(0.00)
	[0.00–2260000000]	[0.05–0.36]
Hours vary	0.96	0.90
	(0.93)	(0.45)
	[0.36–2.59]	[0.69–1.18]
Work city	n.c	2.47
		(0.07)
		[0.94–6.50]
Work outback	n.c	0.77
		(0.62)
		[0.28–2.14]
Hospital work	0.43	0.19
	(0.07)	(0.00)
	[0.17–1.08]	[0.10–0.37]
Co-location	1.80	1.24
	(0.07)	(0.29)
	[0.95–3.43]	[0.83–1.86]
Immigrant	1.54	0.53
	(0.65)	(0.49)
	[0.23–10.1]	[0.09–3.14]
Number of children	0.48	0.26
	(0.20)	(0.00)
	[0.16–1.46]	[0.18–0.37]
High income	0.43	0.24
	(0.17)	(0.00)
	[0.13–1.44]	[0.13–0.45]
Low income	2.18	7.71
	(0.05)	(0.00)
	[1.01–4.71]	[5.09–11.7]
Leave direct care	1.80	2.49
	(0.00)	(0.00)
	[1.55–2.09]	[1.95–3.19]
Leave medicine	1.72	2.30
	(0.00)	(0.00)
	[1.49–1.98]	[1.88–2.81]

*n.c.* regression did not converge, *n.o.* no observations

## Abbreviations

GP: General practitioner
MABEL: Medicine in Australia Balancing Employment and Life
OR: Odds ratio

## Footnotes

1 As is standard in studies of health care professionals [3, 6, 21], individuals who change practice or employer while continuing to perform GP work are not classified as engaging in turnover, while retirements are so classified.

2 Cut-offs of 25 h for part-time employment and children < 3 years of age were considered but rejected due to small cell sizes.

3 Since 2008–2015 was a time of economic turbulence, GP income was linearly regressed against year dummy variables, and the standardized residuals utilized to create the percentile cut-offs.
